# Odontological Society of Pennsylvania

**Published:** 1891-05

**Authors:** 


### ODONTOLOGICAL SOCIETY OF PENNSYLVANIA.

(For paper of Dr. Chupein, see page 213.)

*Dr. F. M. Dixon.*—I must take exception from beginning to end to Dr. Chupein's paper, and I do it with all kindness.

This question, Mr. President, I thought had been talked to death, was dead and buried; not because it was unimportant, but because I believed dentists had made up their minds that it was a subject requiring an amount of judgment which every man does not possess, whether the sixth-year molar should be extracted or whether it should not be; or whether it should be extracted at a certain period. Now, my impression with regard to this tooth is—I say it honestly—that it is one of the most important members of the human mouth. It is the broadest on its grinding surface, and it occupies the central position among its fellows.

Why should it be extracted when there are teeth in the mouth that are not worth one-fourth what the sixth-year molar is, simply because the grinding surface is such that they cannot be brought into place as the sixth-year molar can? We cannot always judge whether we should keep it there until all the teeth are in place and the child, or young man or woman, has grown to maturity, for it sometimes happens that when the sixth-year molar is extracted the teeth do not come together.

Not in one solitary instance do I recollect having lost a first molar in practice, which now, I am happy to say, I never think of extracting. The difficulty in saving this tooth is that it is not taken in time. If I fill a sixth-year molar when the cavity is small, and the filling should fail, it is a serious matter to be obliged to fill it again. As it is the most useful tooth, is it not worth the trouble? If sometimes it is necessary to extract a tooth, why must we take the best and most useful in the mouth instead of one less important? Very often the cervical margin of the first molar and second bicuspid come together more tightly than at the upper surface. Then I say, if necessary to extract a tooth, why not take the second bicuspid? and then will be left a perfect set of teeth.

I should like to hear from some of the other members.

*Dr. G. L. S. Jamison.*—I feel that it would be rather a difficult task for me to speak at length after Dr. Dixon, and I feel rather out of place. Of course my experience has been more limited than his, but I might say that, limited as it is, I quite agree with what he has said. I firmly believe that the sixth-year molar is one of the most useful teeth.

This afternoon about the last patient I saw in my office was the case of an improper extraction of a sixth-year molar, causing a deformity of the young man's face. He called my attention to it. He said that the left side of his face was much fuller than the right side. I examined the mouth and found that the sixth-year molar on the right side had been extracted, and that the teeth had grown in such a manner that occlusion was not by any means perfect. Altogether the young man could not experience any satisfaction, and naturally avoided chewing on that side of the mouth, and, as he was a rather good liver, I presume the muscles on the left side were unnaturally developed, causing a deformity in the young man's expression.

If this tooth is to be extracted, it should be done before the eleventh year if the circumstances are such as to warrant it, for the simple reason that if it is left in position until the eleventh year it will soon be followed by the twelfth-year molar, and already probably this molar has made such progress that its position will not be directly in the place of the sixth-year molar, as it would be had the latter been extracted when the patient was possibly eight or nine years of age.

The essayist laid great stress on the fact that the sixth-year molar was the cause of general decay. Now, that has not been my experience, and it seems to me that if a patient is put in your hands before he is eleven years of age, that by filling and refilling, if necessary, this tooth can be kept in position and can be made serviceable and can be saved as long as any of the other teeth in the mouth. I have seen a great many of Dr. Dixon's patients, through his kindness from time to time. I don't believe that he has in many cases ever advocated the extraction of the sixth-year molar, except when he could not possibly save it by filling.

As far as I am personally concerned, from my limited experience, I would say that it is better to sacrifice either the first or second bicuspid rather than the sixth-year molar. Of course it is a subject that requires great judgment, and there are many circumstances to take into consideration, but I am in favor, on the whole, of retaining it and sacrificing some other tooth.

*Dr. E. C. Kirk.*—If I apprehend Dr. Chupein's position exactly, I understand him to advocate the extraction of the sixth-year molar in a majority of cases as a prophylactic measure against the decay of other teeth, and claims the advantage that decay by contact with other teeth is thus avoided.

In general I am thoroughly opposed to the practice of extracting the sixth-year molar for such purposes. It does not seem to me to be the right line in which to expend our energies towards obtaining prophylactic results, so far as decay of the teeth is concerned. I see no reason why the sixth-year molar should decay sooner than the other teeth except that it is exposed to action longer than any other tooth, and if it is properly taken care of there is no reason why it should be more vulnerable than others. I believe the gum margins in such cases more than balance any good we get from the retardation of the process of decay. Therefore, as a prophylactic measure, I would be opposed to it. Of course there are individual cases where the sixth-year molar could be extracted to advantage; cases of peculiar irregularity where the teeth are so damaged that it should be taken out the same as any other tooth, and not because it is the sixth-year molar.

I thoroughly agree with Dr. Dixon that it is the most valuable tooth in the denture. I think I have nothing more to offer on the subject.

*Dr. L. Ashley Faught.*—Some years ago, at a meeting of the Pennsylvania State Dental Society, I read a paper on "Oral Hygiene." The gentlemen gathered at that meeting were very much on the lookout for mistakes, and some of the papers that were read were pretty severely handled, and when my turn came to read my paper, I had considerable doubt as to whether I would read it or not, considering the fact that I had taken the opportunity of saying something in regard to the sixth-year molar. I thought it might possibly prove a point of attack for those who were looking for victims; but I had a redeeming clause in it, it seems, and they let it alone, and that redeeming clause was: I would extract the sixth-year molar *for cause;* and that seemed to save my head at that time. Nevertheless, I am surprised that we should discuss to-night the matter of the extraction of the sixth-year molar, and that any one who has had an opportunity of viewing mouths and examining diseases of the teeth should desire the retention of this tooth in all cases. The only thing that I can say for those who favor its preservation under all circumstances would be that they have not examined mouths where the sixth-year molar had been extracted. If they had had an opportunity of seeing this extraction done as I believe it ought to be done, I think they would feel the same as I do in regard to it,—that it is a benefit.

I must say that I do not believe in the wholesale retention or removal of the sixth-year molar in all cases, under all conditions, and under all circumstances, but still adhere to my original statement,—for cause. I think there are some mouths where it would be highly injudicious, and I think many times it is the best thing for the patient.

I have seen case after case in my own practice where the removal of the sixth-year molar was indicated, as when the twelfth-year molar is beginning to make its eruption. I believe that if the sixth-year molar is removed at that time, under almost all conditions, unless there be some other mechanical influence exerting its power upon the tooth, that the twelfth-year molar will come forward without turning or disturbing the normal occlusion of the teeth.

That is my position in regard to the sixth-year molar. I believe it to be sound practice, and believe that great good can be done to our patients.

(Question asked as to what is meant by the clause *for cause.*)

It is hard to describe to another those particular things which you would recognize. I am in the position of the judge who gave his timid young friend coming to the bench this advice: You are going to be called on to give your opinions; in doing this, never give your reasons, because in nine cases out of ten your opinions will be right and your reasons wrong.

With judgment, care, and watchfulness, we recognize that under certain conditions certain things are intended, and we know that with due attention good results will follow. It is impossible to transmit that acute knowledge from one person to another.

*The President.*—Give a typical case.

*Dr. Faught.*—Well, if I had a sixth-year molar largely broken down in teeth that would have a tendency to disintegration, showing a weak structure, coming on to that age of life in which perhaps one or two of them are involved in trouble, I should have no hesitation in removing the sixth-year molar. I believe that in such a case the extraction of the sixth-year molar will give you, instead of a bicuspid and small wisdom tooth in the arch, which is of very little use, a twelfth-year molar in good position, and the other molars will also develop and come forward to do the same service.

*Dr. S. E. Gilbert.*—The only thing I can say is that a man must use his brains. There are cases that I have seen where the extraction of the sixth-year molar, and also its retention, was very beneficial.

I have a case in mind now where the extraction of the first permanent molar has run through a whole family. I cannot express the terms that that family can use in disapproval of it, for the reason that it was extracted at the wrong time and the teeth stand in irregular position.

As Dr. Kirk has said, there is more injury to the gum margins than will be benefited by removal. I do not believe in separation. I have had considerable to contend with from teeth having been separated by filing, and if there is any nuisance in a man's mouth it is that. I do not like to say anything against another man,—in fact, I will not of a legitimate practitioner,—but it is pretty hard work to smooth over this mode of practice.

I do not believe in the wholesale extraction, neither in the wholesale retention. You must use your judgment. There are some cases where, if they are removed, it will save an immense amount of trouble afterwards. In my own mouth I believe the denture would be in great deal better shape if the sixth-year molar had been removed at the proper time.

*Dr. Rehfuss.*—I am pleased to hear this paper of Dr. Chupein's, and I feel gratified to have heard the different speakers condemn the extraction of the sixth-year molar as an invariable rule. Of course it is recognized, as the previous speakers have said, that at times its judicious extraction is a benefit, but it is difficult to recommend this operation.

The sixth-year molar is understood to be the connecting link between the first and second dentition, and I think it would not only be malpractice, but wilful malpractice, for any one to presume to extract the sixth-year molar previous to the twelfth year; because we know that between the seventh and ninth year, or even as late as the eleventh year, the sixth-year molars are the only teeth that are in place for masticating. It brings the anterior teeth in occlusion, and deformity results,—unnatural pressure on the upper teeth by the lower, which causes protrusion of the upper teeth.

Now, the authorities that favor the extraction of the sixth-year molar say that the time to do it is just previous to the appearance of the twelfth-year molar, and that prevents tipping; but when this is not done we should let it alone until the eruption of the wisdom teeth, so the space will close up.

In addition, I might say that I disagree with some of the authorities that the sixth-year molar, in texture and quality, is inferior to the other teeth in the mouth. That is, from a limited experience; but still I have noticed that the second bicuspid decays oftener and more frequently than the sixth-year molar; and I also agree on that point with the previous speaker, that if any teeth are to be extracted, I would sacrifice the second bicuspid in preference to the sixth-year molar.

*Dr. Gilbert.*—I have a son who is not eleven years old, yet the twelfth-year molars are thoroughly formed, and they have half their length exposed. I think it would be rather late to extract. I think Dr. Faught's idea of extraction is nearer the proper time.

*Dr. Rehfuss.*—I think it ought to be extracted previous to the twelfth year. In speaking of the twelfth year, I was speaking of the authorities who are in favor of extracting the sixth-year molar.

*Dr. Kirk.*—I would like to ask if any one has ever seen a case where the extraction of the sixth-year molar does not make a difference in the appearance of a set of teeth, and if it could not always be told that a tooth had been extracted by the appearance of the space and the gum margins, etc.? I have never seen a mouth where the tooth has been extracted and the space closed up, but that the gum festoon looked different. There is also nearly always some little tilting forward of the twelfth-year molar.

I have pursued, at one time, the extraction of the sixth-year molar as a prophylactic measure until I convinced myself that I was on the wrong track. I have tried it, and I speak of it as the result of my experience in my own work and what I have seen in the mouths of patients who have come from other dentists; and where I extract now it is for the correction of some special irregularity, not with the belief that it will prevent caries; and for the first reason I extract the second bicuspid as frequently as the sixth-year molar.

*Dr. Chupein*—I have observed it more in the lower jaw than in the upper. I have seen it in the upper jaw where it would be impossible to detect whether the first molar had been extracted.

I would say that I rather object to waiting as long as Dr. Faught did for the eruption of the second molar pushing through the gum before permitting the first molar to be extracted, and I think it would be preferable, as soon as we notice a little swelling, indicating that the tooth had not yet quite protruded, to extract it, rather than wait until it has come through the gum thoroughly; but if you extract it before that time it will come forward until it will nearly occupy the position of the first molar.

*Dr. Ambler Tees.*—I must say that I approve a great deal of Dr. Chupein's paper. Many years ago I tried to save these teeth, and filled and refilled, and patched up the mouths of my patients, but for many years I have changed my tactics, and it has been my practice to order the extraction of the sixth-year molar, when proximal decay is much advanced, at eleven years of age, and I do this at this period to avoid the tilting that has been spoken of by several gentlemen.

For instance, if the lower molar shows proximal decay, I order its extraction at the age mentioned, and I also order the extraction of its antagonist, whether it indicates decay or not. I do this to give perfect occlusion.

Several years ago a young patient from one of the boarding-schools was sent to me to have two devitalized lower molars treated; the crowns were half decayed away. She was a young lady about sixteen years of age. From my experience I knew that those teeth would have to come out, and that in a very short time. One of the lady teachers at the school came with her. She said, "Now, doctor, we have been ordered by the dentist in New York to come to you for the treatment of those teeth. He has been treating them for some time, and recommended us to you to continue it." I told her then that my conscience would not allow me to continue the treatment; that I would either have to send the patient back, which was almost impossible, or else refuse the case. The daughter said, " We have been sent to you, and now we are going to let you do as you think best." I told the young lady that the best thing to do was to have the teeth taken out. She said she wanted to place herself in my hands, and she insisted on my doing the work. This I did, and treated and filled the other teeth. The first of July I sent my bill to her father, and on returning from my vacation I found a letter from him, censuring me very much, and said that he had never paid a bill so reluctantly. He represented that I had extracted teeth by the wholesale for his child, and that he would never place her in my hands again. I simply wrote to him that I had done my conscientious duty, and asked him to make a careful examination of the young lady's mouth after two years and write and tell me whether he could discover that any teeth had been extracted. I have not heard from him, though that has been four or five years ago, and I know that there will be no spaces between either the upper or lower teeth. The twelfth year molar will come up to the bicuspids, showing a case like Dr. Chupein exhibited of the approximation of all the teeth.

*Dr. A. W. Deane.*—In 1879 or 1880 I heard you, Mr. President, with some of the others, advocate the retention of the sixth-year molar at the Pennsylvania Dental College. From that time on I have thought best to save all the teeth that God has put there, unless it be the wisdom tooth.

I have under my care about two hundred girls, and have had for six or seven years, of the St. Joseph's Home, at Seventh and Spruce Streets, and I would be very glad to have any dentist go there and use my name and examine any of the mouths, and see if they can find a mouth where they think the extraction of the sixth-year molar would be beneficial. I work just as hard, perhaps a great deal harder, to save a sixth-year molar than almost any other tooth, unless it be a lateral. A great deal of trouble is experienced with the first permanent molar, yet I cannot see where it is beneficial to extract the tooth; as these gentlemen who advocate its removal say that they cannot tell you when nor why to extract it, and Dr. Faught says there is just one time to remove it. What is that time? He cannot tell us. Dr. Chupein says extract it, but why? I do not see this shown in the case he passed around. We find many teeth that do not touch each other in the mouth anywhere at all, yet there are cavities in the proximal surfaces. Why is this?

In my own mouth the sixth-year molar was extracted when I was quite young. My mother took me to the dentist, and I had the tooth removed in the lower jaw, and the twelfth-year molar is tipped over, and if the man who extracted it were to receive all the maledictions I have passed on him, from food getting down and pressing the gum and causing the pain I have suffered, he would not need any Hades hereafter. The other sixth-year molar was extracted while I was in college, and that bothers me quite as much.

*Dr. C. H. Littleton.*—I think the extraction of the sixth-year molar very bad practice. Its strong support to the arch and its broad surface, so useful in masticating, are of sufficient importance to command our best skill in its preservation. I believe the advocates for the extraction of this tooth usually recommend it when the teeth are soft and much crowded in the arch. In such cases I have entirely satisfactory results by extracting the second bicuspid above and below.

My predecessor, Dr. Gates, had under his care three brothers who married three sisters. In these families there were twenty-eight children. All of their teeth were very soft, with a great tendency to crowd in the arch. When they came into my hands, Dr. Gates had extracted the second bicuspid above and below for eleven of them. Since that time I have had the same operation performed for eight more. In every case (except one which I have not seen) the spaces are entirely closed without the slightest irregularity of the teeth. Heavy approximal pressure is undoubtedly the cause of much decay in soft, chalky teeth. In such mouths, often, the approximal surfaces of the bicuspids are flattened, admitting of decay when it would not otherwise occur. The first molar will harden with age, and I have generally found it much easier to save than the second bicuspid.

*Dr. W. G. A. Bonwill.*—I rather compliment this gentleman on having the courage to come here and read a paper of that kind, but I am not with him; I compliment him, nevertheless, for his effort; but if I can raise my hand or voice to convert him, I would willingly spend an hour or the whole evening. It is very hard for a dentist to practise thirty years without having made some mistakes. Show me a man who has made no mistakes, and I will show you a man who has done nothing.

I do not agree with my friend. I do not see any way of settling this question or settling any of the difficulties that exist. We have no system of dentistry, although we have a number of volumes said to be the American System of Dentistry, but who can practise by it? Can we give these as a safe guide to the beginners in the profession? Can we give them as a complete guide? Can we go to any one individual man in this assembly and get from him that which would suit us in practice on all occasions?

There is no system as yet, and I am very glad he has attempted to make one for saving the teeth. I have said of others, where they are using all amalgam or all plastics, if they can save teeth the way they do and cannot save them in any other way, God speed to them.

How are we to get clear of all these things? This gentleman comes here and tells us why he does it, and I believe he is a very honest man and speaks from his convictions, but still, at the same time, I would like to tell him just where he is doing wrong.

I have done a great deal of extracting for people who had not the money and time to have their teeth filled, and I have seen decay as the result of this extraction. I have had several cases in my office from other and older dentists, who very early in life extracted the sixth-year molar with most happy results, so far as the second molar coming forward and filling up the space.

When you come to take into consideration how nicely a crown can be put upon a root of the first molar, and serve the purposes of mastication for years, there is hardly any occasion for extracting.

I find it really the most important tooth in the mouth when the mechanical structure of the mouth is considered.

Of all the cases that have come to me from the hands of others where the first permanent molars have been extracted, I have yet to see, except in a few individual cases, where it would have stopped caries. Extraction will cause the rest of the teeth to fall back in the arch.

The first case I had was a gentleman whose teeth were so decayed it was impossible for me at that time to preserve them. All of his lower teeth were badly gone on the approximal surface at the age of twenty-one. I had to remove from first bicuspid the whole enamel. It was pitted as if it had had the small-pox. I left nothing but a cone standing upon its base. I cut off the entire distal surface of that tooth. For thirty-three years that gentleman has been coming to me, and he has never lost one of the lower teeth. The sixth-year molar is there, and he never had a tooth out until about three weeks ago. And all the upper teeth gone at the age of twenty-one. The first bicuspid, without a trace of enamel upon it, remained in that condition for thirty-three years. All at once it commenced to show signs of decay above the edge of the cervical border; I filled it. The next year another place commenced at the very top of the cone. I filled that, and I have been watching it with the greatest solicitude and care ever since. So, when I look back and see the success of that one case, and how much could be cut from the surface and still save, why, sooner than extract a molar now, I would cut every particle of its enamel off and put a crown on. Besides, as I said a moment ago, crowning the teeth is such an important thing that for that reason alone I would not do it.

I want to propose one thing here to this Society. I think the best way of getting at this thing is to measure somebody else's half-bushel by our own. There are many who have cases of children, and they are generally willing to come before societies. If we could have a fair understanding with each other and with our patients, we could show these things. I am willing to bring my own children, and plenty of my patients would do it. I think I am right in this line of work. That is the best way to prove it.

Dr. Chupein brings this as a representative case [referring to model], but if my experience is worth anything, and if contour work is worth anything, then I beg to disagree with him; for if it is a representative case showing the possibility of saving teeth, it won't do it. The approximal surfaces of the bicuspids are safe, but the distal surface of the second bicuspid would go in spite of all efforts.

*Dr. James Truman.*—This discussion reminds me of one fact,—that all things move in circles, for I believe that these subjects come up regularly every one or two years.

I have been pleased to find that quite a number have defended Dr. Chupein, for I feared at one time that he was doomed to be in the minority of one or two; forty years ago he would have been in the majority. At that time nearly all the sixth-year molars were extracted. They were condemned with undue haste and without proper consideration.

I remember twenty years ago of having had a discussion on this very topic with my colleague and friend, Dr. Barker. The discussion grew very warm. He said to me, during the meeting, that he would bring two children with him the next day, and he would convince me that the extraction of the sixth-year molar was the proper thing to do. He brought his two children, and they were examined by others as well as myself. Beautiful sets of teeth they were, but in each of them the sixth-year molar had been removed, as he had stated. I then told him that if I lived to be old enough to see these children when grown up, that they would exhibit spaces between all these teeth anterior to the sixth-year molar. He regarded the statement with incredulity. I have never seen them from that time to this, but a friend of mine who has operated for the family since the father's death assures me that the spaces are there.

What is the position of this sixth-year molar? It occupies a place in the series more important than any of the posterior teeth. It is the key of the entire arch. The direction of the anterior teeth is towards the median line. This inclination is essential to perfect articulation. The removal of any of the teeth changes this position to the direct vertical, and the disturbance will be in proportion to the size of the tooth. The removal of this first molar utterly destroys perfect articulation and makes spaces difficult or impossible to remedy.

Now, what is the sixth-year molar, that it should be condemned? It is not, of course, as dense at the age of six years as the twelfth-year molar is when it comes in; but it is demonstrable that every tooth increases in density as the years go by, and the tooth becomes more resistant to caries each added year.

I do not see how our friend Dr. Chupein can take, at this advanced period, the position he has to-night. It seems to me to be retrograding to that period I alluded to; but I think the profession has arrived at a time of increased intelligence in regard to the salvation of the teeth than would seem to be the case by the paper that has been read this evening. We are here to save teeth; that is our business, and we are not extractors of teeth, whether these be the sixth-year molars or others in the series. We must save them to the very last root, if possible.

*Dr. Chupein.*—It is not the object of my paper to recommend the extraction of all sixth-year molars, but only to take it in the condition that we find it and extract them when we think it is necessary. I have very frequently in my practice saved all the teeth that came to me.

I know of some families that have been sent to me, and, after giving the attention required, I have not seen them for a long time, and have wondered whether they were dissatisfied with my treatment and have gone elsewhere; but eventually some of these parties would return, and examination would show that other teeth had been extracted. I knew that I had not removed these, and I came to the conclusion that their teeth were in better condition with the teeth that had been extracted than if they had remained with me and I had attempted to save them; so that I cannot be classified as an extractor of teeth.

So far as the spaces that are referred to by Dr. Kirk are concerned, I do not think that I have observed that the gums were ever inflamed by the spaces left between the teeth, such as represented on that side of the model. [Indicating.] I do not mean to say that teeth cannot be plugged and replugged again and again; but then I think we owe some consideration to the patients that come to us, and should try to save them as much pain as we can. I consider it good practice to extract this tooth at certain times.

*Dr. Truman.*—We all owe Dr. Chupein thanks for bringing this paper before us for discussion.

